# Effects of recombinant human growth hormone on HIV-1-specific T-cell responses, thymic output and proviral DNA in patients on HAART: 48-week follow-up

**DOI:** 10.1186/1476-8518-6-7

**Published:** 2008-10-31

**Authors:** Anna A Herasimtschuk, Samantha J Westrop, Graeme J Moyle, Jocelyn S Downey, Nesrina Imami

**Affiliations:** 1 Department of Immunology, Imperial College London, Chelsea and Westminster Hospital, 369 Fulham Road, London, SW10 9NH, UK; 2 Department of HIV/GU Medicine, Imperial College London, Chelsea and Westminster Hospital, 369 Fulham Road, London, SW10 9NH, UK

## Abstract

**Background:**

Efficacious immune-based therapy in treated chronic HIV-1 infection requires the induction of virus-specific CD4^+ ^T cells and subsequent maturation and maintenance of specific memory CD8^+ ^T cells. Concomitant daily administration of recombinant human growth hormone (rhGH) with highly active antiretroviral therapy (HAART) was used in chronically infected patients with lipodystrophy in an attempt to reconstitute these virus-specific T-cell responses.

**Methods:**

Individuals with chronic HIV-1 infection on HAART were enrolled on a randomized, double-blinded, placebo-controlled study to receive rhGH therapy. We assessed HIV-1-specific proliferative CD4^+ ^and interferon-gamma (IFN-γ)-producing CD8^+ ^T-cell responses, quantified thymic output and proviral HIV-1 DNA at the following time points: baseline; after 12 weeks of rhGH therapy; at 24 weeks, after randomization into three groups [placebo weeks 12–24 (Group A), alternate-day dosing weeks 12–24 (Group B), and twice-per-week dosing weeks 12–24 (Group C)]; and at 48 weeks after all patients had received HAART alone for the final 24 weeks.

**Results:**

We found significant increases in both proliferative CD4^+ ^and IFN-γ-producing CD8^+ ^HIV-1-specific T-cell responses after daily administration of rhGH. This increase was focused on HIV-1 Gag-specific T-cell responses. Following subsequent randomisation into different dosing regimens, HIV-1-specific proliferative CD4^+ ^T-cell responses declined in patients receiving less frequent dosing of rhGH, while virus-specific IFN-γ-producing CD8^+ ^T-cell responses were maintained for longer periods of time. There was no significant change in thymic output and the cell-associated HIV-1 DNA remained stable in most patients. An increased anti-HIV-1 Nef-specific CD4^+ ^T-cell proliferative response was correlated to a decrease in proviral load, and an increased HIV-1 Gag-specific IFN-γ-producing CD8^+ ^T-cell response correlated with an increase in proviral load.

**Conclusion:**

The implication of these data is that daily dosing of rhGH with HAART, in addition to improving HIV-1-associated lipodystrophy, may reverse some of the T-lymphocyte dysfunction seen in most treated HIV-1-positive patients, in a dose-dependent manner. Such immune-based therapeutic strategies used in treated, chronic HIV-1 infection may enable the induction of virus-specific CD4^+ ^T cells essential for the subsequent 'kick-start' and expansion of virus-specific CD8^+ ^T cells.

**Trial registration:**

GH in Lipoatrophy IMP22350.

## Background

Infection with HIV-1 causes a severe down-regulation of virus-specific CD4^+ ^and CD8^+ ^T cells that is not restored upon treatment with highly active antiretroviral therapy (HAART). The aims of immune-based therapeutic interventions in the presence of HAART are to deplete viral burden in cellular reservoirs, to induce and maintain virus-specific responses, and to facilitate regeneration of the immune system; thereby allowing the HIV-1-infected individual to control viral replication and opportunistic pathogens in the absence of drug therapy [1,2]. One candidate molecule to include as part of such an intervention is growth hormone (GH). GH exerts stimulatory effects on different cells of the immune system, mediated either directly or indirectly through insulin-like growth factor-1 [3-5], and has implications in T-lymphocyte development and function [6]. This suggests a role for recombinant human growth hormone (rhGH) as a possible immunomodulatory therapy, complimentary to the benefits of effective antiretroviral drug therapy, for HIV-1 infection [5]. Furthermore, studies in both HIV-1-infected adults and adolescents with lipodystrophy show impaired GH secretion [7,8]. The use of rhGH for the treatment of HIV-1-associated wasting syndrome demonstrates its suitability for routine clinical care [9,10].

The generation of fully functional virus-specific peripheral CD4^+ ^and CD8^+ ^T lymphocytes in treated chronic HIV-1 infection is of considerable importance [11,12], and may be critical for enabling control of viral activity and retarding disease progression in persistent HIV-1 infection in the presence of, and possibly following subsequent removal of, HAART [13]. The success of immune-based therapies will depend on full restoration of numbers and function of the CD4^+ ^helper T lymphocytes (HTL), antigen presenting cells (APC) and CD8^+ ^cytotoxic T lymphocytes (CTL) at all stages of disease [14]. Although successful induction of HIV-1-specific T-cell responses has been observed with various immunotherapeutic approaches in the presence of HAART [13], the major drawback has been that such responses were transient; indicating that eradication of virus presents a difficult therapeutic goal. Generation of activated virus-specific CD4^+ ^HTL, which may be preferentially targeted by HIV-1, also presents the risk of *de novo *infection and clonal deletion [15]. Therefore the adverse effects of HIV-1 should be taken into account when immunotherapy is used to induce such responses. Nevertheless, induction of HIV-1-specific T-cell responses in HIV-1-positive individuals comparable to those observed in long-term nonprogressors [2,16,17], remains of paramount concern.

We assessed changes in T-lymphocyte function (proliferation and IFN-γ production), thymic output and proviral HIV-1 DNA in twelve HIV-1 infected individuals on long-term successful HAART who received rhGH therapy for lipodystrophy. Our data provide evidence that daily administration of rhGH for 12 weeks dramatically increased HIV-1-specific CD4^+ ^HTL and CD8^+ ^CTL responses. This was reflected by an expansion in HIV-1-specific CD4^+ ^HTL proliferative responses directed to Gag, as well as to the HIV-1 immunogen Remune™, and its 'native' p24. Responses to recombinant vaccinia virus (rVV) constructs and overlapping peptides spanning the HIV-1 proteins Gag and Pol were carried out using IFN-γ ELISpot analysis to characterise HIV-1-specific CD8^+ ^CTL responses. Whilst reduction in dosing over a further 12 weeks resulted in the loss of virus-specific CD4^+ ^HTL, the virus-specific CD8^+ ^CTL responses seen at week 12 were sustained by week 24, and gradually declined by week 48. Levels of T-cell receptor excision circles (TREC) and proviral DNA remained constant throughout in the majority of patients. Increases in proviral DNA were observed in only 3/12 patients. An increased anti-Nef proliferative response was correlated to a decrease in proviral load, and an increased anti-rVV Gag IFN-γ response correlated with an increase in proviral load, suggesting that administration of rhGH with HAART may partially reverse some of the damage exerted on the immune system by HIV-1.

## Materials and methods

### Study subjects and samples

Blood samples were taken from twelve HIV-1 infected patients with lipodystrophy receiving HAART (9 on NNRTI and 3 on PI based regimens) for >4 years. Mean age ± sem was 43.4 ± 2.1 years, viral load was undetectable in 83% (10/12) of patients, and absolute mean ± sem CD4^+ ^and CD8^+ ^T-cell counts were 478.4 ± 55.6 cells/μl and 1020.0 ± 15.6 cells/μl blood respectively (Additional file 1). rhGH was administered to all patients for 12 weeks at 4 mg/day (Serostim, Serono International, Geneva, Switzerland). This was followed by randomisation into three groups: (A) receiving placebo, (B) alternate-day dosing, or (C) twice-per-week dosing of rhGH which continued for a further 12 weeks (i.e. week 24 of the study); after which patients went back to receiving HAART alone (no immunotherapy). Thus samples were collected at baseline, weeks 12 and 24 of the study plus a follow up visit at week 48 from the start of the study. The patients' informed consent and Ethics Committee approval were obtained for the studies described.

### Plasma viral RNA assay

Viral load in patient plasma was measured at each time point of sample collection using the Versant HIV-1 RNA 3.0 branched DNA assay (lower detection limit of 50 copies/ml plasma, Siemens Healthcare, Camberley, UK).

### Antibodies, flow cytometry and lymphocyte subset quantification

Murine, anti-human monoclonal antibodies (mAb) to CD3, CD4, CD8 and CD45 (TetraOne, Beckman Coulter, High Wycombe, UK) were used to mark lymphocyte subsets within whole blood and then evaluated using a Cytomics FC 500 flow cytometer (Beckman Coulter) and Tetra CXP (version 2.2) software.

### HIV-1 antigens, peptides and recombinant vaccinia vectors (rVV)

HIV-1 recombinant antigens and peptides were obtained from the Medical Research Council Centralised Facility for AIDS Reagents (NIBSC, Potters Bar, UK), and used as previously described [17]. Cells were cultured with antigen at final concentrations of 10 μg/ml. In addition, the inactivated, gp120 depleted, HIV-1 immunogen (Remune™), and its constituent Gag-p24 antigen ('native' p24: clade G) (both from Immune Response Corp. Carlsbad, San Diego, CA) were used at 3 μg/ml [18]. The 22 Gag p24 peptides were 20-mers with a 10 amino acid (aa) overlap covering p24 Gag (aa 133–363 of HIV-1 SF2, ARP 788.1-.22), and were used in a pool of 22 at a final concentration of 4 μg/ml each. The 110 Pol reverse transcriptase (RT) peptides were 15-mers with a 10 aa overlap covering RT (HIV-1 LAI, ARP 7010.1-.110), and were used at a final concentration of 1 μg/ml each in a pool of 110. The recombinant vaccinia virus (rVV) constructs were obtained through the NIH AIDS Research and Reference Reagent Program (Rockville, MD, USA) and from the MRC AIDS reagent project (NIBSC) and encoded Gag, Pol or PB2 (influenza A virus basic polymerase 2 subunit (generous gift of G. Smith); negative control) proteins.

### Proliferation assays

PBMC (10^5^/well) in 10% AB plasma/RPMI (200 μl; Sigma, Poole, UK) were cultured in triplicate with either antigen, mitogen or cytokine in round-bottomed microtiter plates (Greiner, Stonehouse, UK). Antigens, mitogens and cytokines were used as described previously [17,19]. On day 5, each well was pulsed with 1 μCi ^3^H-methyl thymidine (^3^H-TdR; Amersham International, Amersham UK) and 16 hours later cells were harvested onto glass fibre filtermats (Wallac Oy, Turku, Finland). Proliferation, as measured by ^3^H-TdR incorporation, was evaluated by liquid scintillation spectroscopy using a 1205 Betaplate counter (Wallac). Control wells, for calculation of background activity, contained PBMC only. Results are expressed as stimulation index (SI) scores, which were evaluated as the experimental result divided by the background result. A positive response is defined as an SI score of 5 or more.

### Overlapping-peptide based ELISpot assay for enumeration of IFNγ-producing virus-specific CD8^+ ^T cells

PBMC at 2.5 × 10^5 ^cells/well were added to 96 well polyvinylidene difluoride (PVDF) backed plates (MAIP S45; Millipore, Bedford, MA) that were previously coated with 100 μl of anti-IFN-γ mAb 1-D1k (10 μg/ml; Mabtech, Stockholm, Sweden) and incubated overnight at 4°C. Peptide pools or phytohaemaglutinin (PHA; positive control) at a final concentration of 10 μg/ml were added directly to wells in 100 μl of RPMI. Negative controls comprised cells cultured in absence of peptide and were always <4 spot forming cells (SFC) per 2.5 × 10^5 ^input cells. Plates were incubated at 37°C, 5% CO_2 _for 16 hours and then processed as described previously [17,19]. Spots were counted under magnification (×20) with a Wessex Stereomicroscope (Southern Microscopes, Maidstone, UK) and confirmed on the Zeiss KS ELISpot system (Imaging Associates, Thame, UK). Responses were considered significant if a minimum of 5 SFC were present per well once background was subtracted and the result was at least double the background. Data represent mean values of duplicate wells at each point and variation among duplicates was <10%.

### Recombinant vaccinia virus construct based ELISpot assay for enumeration of IFN-γ-producing virus-specific CD8^+ ^T cells

Recombinant vaccinia virus constructs expressing either HIV-1, Gag or Pol protein were used for *in vitro *infection of PBMC used in the ELISpot assay. Previous studies have demonstrated that CD8^+ ^T cells produce >90% of the IFN-γ release in response to rVV stimulation of PBMC [20]. PBMC were infected with rVV constructs for 1 hour at 37°C in 5% AB plasma with 20 infectious units per cell of rVV Gag, rVV Pol and rVVPB2. Cells were washed twice in RPMI containing 5% AB plasma (1800 rpm/5 minutes). Finally, cells were resuspended in RPMI 10% AB plasma and plated out in duplicate, directly onto ELISpot plates at 2.5 × 10^5 ^PBMC per well. PHA was used as positive control and rVV PB2 was used as negative control. Detection and enumeration of SFC was carried out as described above. The mean values from duplicate wells were taken. A positive result is defined as at least twice the background and a score of 5 or more above background, which was always <20 SFC/10^6 ^PBMC.

### DNA extraction and PCR analysis of sjTRECs

Extraction of DNA was carried out from 5 × 10^6 ^PBMC using the Puregene DNA purification kit (Gentra, Flowgen, Staffordshire, UK). PCR amplification of signal joint T-cell receptor excision circle (sjTREC) was carried out following a previously described method [21,22] and PCR products were resolved on 1% agarose gel. A standard curve was used to quantify sjTREC numbers as TREC per 5 × 10^6 ^PBMC.

### Latent proviral DNA: Quantification of HIV-1 DNA in PBMC

HIV-1 proviral DNA was measured using fluorometric PCR methodology as previously described [23,24], with an analytic sensitivity of 10 copies/μg of total cellular DNA. Briefly, HIV-1 DNA levels were assessed from cryopreserved PBMC using the AMPLICOR HIV-1 MONITOR (Roche Molecular Systems, Branchburg, NJ) according to the manufacturer's instructions. All experiments were carried out in duplicate and mean values used.

### Statistical analysis

Computer software (GraphPad Prism^® ^version 5.0, La Jolla, California, USA) was used for all statistical calculations. Analysis of data between patient groups was carried out by the Mann-Whitney test and the Wilcoxon signed rank test was used to compare paired responses from the same patient as appropriate. Data presented as box plots show the median and interquartile range with whiskers representing the 10^th ^and 90^th ^percentiles. Comparisons were carried out between all time points. All statistical calculations, including correlations, were calculated using non-parametric methods. Significance was measured to a 95% confidence interval with p values below 0.05 considered significant.

## Results

Twelve HIV-1 infected individuals, with chronic HIV-1 infection and lipodystrophy, receiving long-term HAART for >4 years entered into a 48-week study. We evaluated T-cell responses to HIV-1, and to other viral and recall antigens, at baseline; after 12 weeks of daily administration of rhGH therapy (4 mg/day subcutaneously); at 24 weeks after randomisation into three groups: (A) placebo (B) alternate-day dosing (C) twice-per-week dosing for weeks 12–24; and at week 48 when all patients had received HAART alone (no immunotherapy) for the final 24 weeks of the study. Patient characteristics at baseline, weeks 12, 24 and 48 are shown in Additional file 1. Baseline viral load was undetectable in 10/12 patients and mean ± sem CD4^+ ^T-cell count was 478.4 ± 55.6 cells/μl of blood and throughout the study viral load remained detectable in only one patient, whilst CD4^+ ^and CD8^+ ^T-cell counts remained unchanged in all patients. CD4^+ ^HIV-1-specific HTL responses were measured using the conventional lymphoproliferative assay, whilst CD8^+ ^HIV-1-specific CTL responses were measured by ELISpot assays using pools of overlapping peptides and rVV constructs.

**Figure 1:**
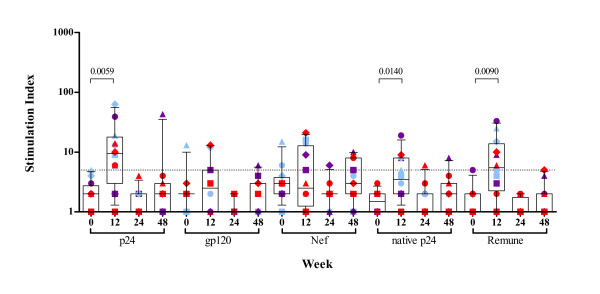
**Proliferative CD4^+ ^T-cell responses to HIV-1 antigens in HAART treated HIV-1-infected patients before and after rhGH immunotherapy.** PBMC from 12 HIV-1-infected patients were cultured in the presence of various HIV-1 antigens in triplicate for 6 days and ^3^H-thymidine incorporation was measured as described in materials and methods. Patient visits are depicted at baseline, weeks 12, 24 and 48. Results are expressed as the mean stimulation index of triplicate cultures with percentage error of the mean <15%. The positive threshold of an SI ≥ 5 is indicated. Box-plots show the median and IQR, and whiskers represent the 10th–90th percentiles. Symbols are specific to each patient according to the key shown in Additional file 1. Randomisation into three groups performed at week 12 is represented by different colours. Group A (red) received placebo, group B (purple) received alternate day dosing of rhGH and group C (blue) received twice weekly dosing of rhGH. Significant p values of <0.05 are shown.

While patients maintained a stable population of cells, at baseline a complete lack of both CD4^+ ^and CD8^+ ^HIV-1-specific T-cell responses was noted in 11 of 12 individuals. After 12 weeks of daily immunotherapy with rhGH in the presence of HAART there was a significant increase in HIV-1-specific CD4^+ ^T-cell proliferative responses; anti-recombinant p24 p = 0.0059, anti-native p24 p = 0.0140, anti-Remune immunogen p = 0.0090 (Figure 1). This increase was focused on Gag-specific and whole HIV-1 antigen (Remune)-specific CD4^+ ^T-cell responses which were positive in 9 of 12 of these patients. CD4^+ ^and CD8^+ ^T-cell counts and viral load remained statistically unchanged (Additional file 1). HIV-1-specific CD4^+ ^HTLs were not maintained at week 24, with less frequent dosing of rhGH, and became undetectable in the majority of patients by 48-week follow up (Figure 1). All figures show the results for all 12 patients, with colours representing the three groups post randomisation.

**Figure 2:**
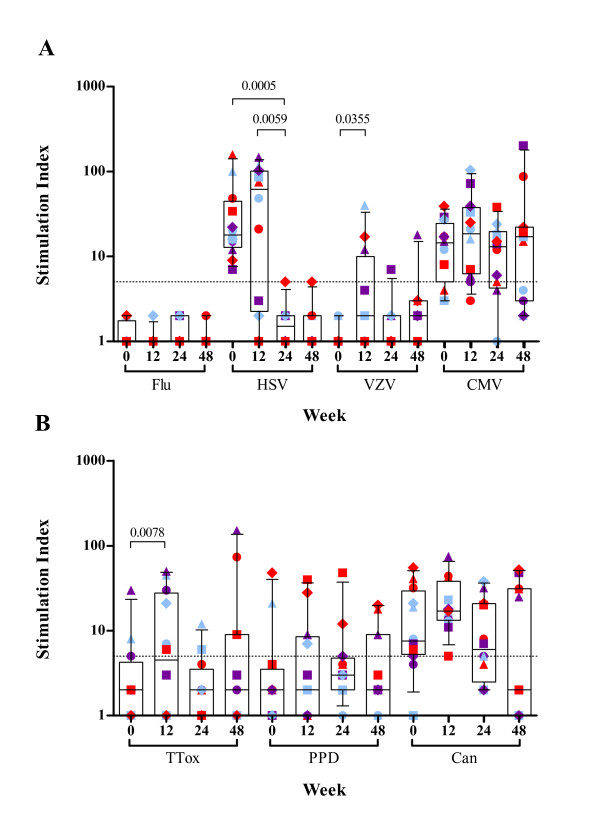
**Proliferative CD4^+ ^T-cell responses to other viral (A) and recall (B) antigens in HAART treated HIV-1-infected patients before and after rhGH immunotherapy.** PBMC from 12 HIV-1-infected patients were cultured in the presence of various viral or recall antigens in triplicate for 6 days and ^3^H-thymidine incorporation was measured as described in materials and methods. Patient visits are depicted at baseline, weeks 12, 24 and 48. Results are expressed as the mean stimulation index of triplicate cultures with percentage error of the mean <15%. The positive threshold of an SI ≥ 5 is indicated. Box-plots show the median and IQR, and whiskers represent the 10th–90th percentiles. Symbols are specific to each patient according to the key shown in Additional file 1. Randomisation into three groups performed at week 12 is represented by different colours. Group A (red) received placebo, group B (purple) received alternate day dosing of rhGH and group C (blue) received twice weekly dosing of rhGH. Significant p values of <0.05 are shown.

**Figure 3:**
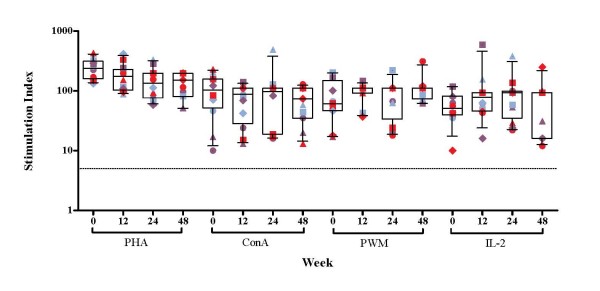
**Proliferative CD4^+ ^T-cell responses to mitogens and IL-2 in HAART treated HIV-1-infected patients before and after rhGH immunotherapy.** PBMC from 12 HIV-1-infected patients were cultured in the presence of mitogens or IL-2 in triplicate for 6 days and ^3^H-thymidine incorporation was measured as described in materials and methods. Patient visits are depicted at baseline, weeks 12, 24 and 48. Results are expressed as the mean stimulation index of triplicate cultures with percentage error of the mean <15%. The positive threshold of an SI ≥ 5 is indicated. Box-plots show the median and IQR, and whiskers represent the 10th–90th percentiles. Symbols are specific to each patient according to the key shown in Additional file 1. Randomisation into three groups performed at week 12 is represented by different colours. Group A (red) received placebo, group B (purple) received alternate day dosing of rhGH and group C (blue) received twice weekly dosing of rhGH.

To address the specificity of the responses generated by rhGH, we also assessed the CD4^+ ^HTL responses to some other viral [Influenza A (Flu), varicella-zoster virus (VZV), cytomegalovirus (CMV)] and recall [tetanus toxoid (TTox), purified protein derivative (PPD) and candida (Can)] antigens. Daily administration of rhGH immunotherapy with HAART had no effect on pre-existing CD4^+ ^T-lymphocyte responses to Flu, CMV, PPD or Can antigens, which was illustrated by the consistent responses directed at these viral and recall antigens throughout the course of study (Figure 2). Lymphoproliferative responses to VZV and TTox were significantly increased upon daily administration of rhGH immunotherapy between time point 0 and 12 weeks (p = 0.0355 and p = 0.0078 respectively; Figure 2). High levels of lymphoproliferative responses to herpes simplex virus (HSV) antigens were observed at baseline and week 12, which were significantly higher than the responses at week 24 where the responses in all but one patient were negative (p = 0.0005 and p = 0.0059 respectively). Anti-HSV responses correlated with clinical manifestations; despite the absence of active herpetic lesions at time of admission, 9/12 patients in the cohort had previous history of herpetic manifestations/complications, accounting for the strong anti-HSV lymphocyte responses observed at baseline and week 12. In addition, immunotherapy with daily rhGH in the presence of HAART had no significant effect on mitogen- and IL-2-induced lymphoproliferative responses, measured in parallel with antigenic responses, over a 12-week period and throughout the course of the study (Figure 3). Daily administration of rhGH also improved body composition (lean body mass) [10].

**Figure 4:**
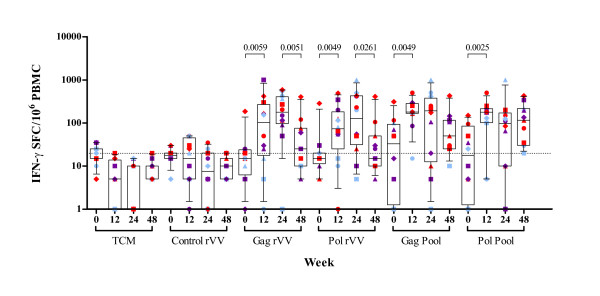
**IFN-γ production by CD8^+ ^T cells in response to rVV HIV-1 constructs and peptide pools in HAART treated HIV^+ ^patients before and after rhGH therapy.** Patient visits are depicted at baseline and at weeks 12, 24 and 48. Results are expressed as the mean number of SFC per 10^6 ^PBMC of duplicate cultures with <10% variation among duplicates. The positive threshold of ≥ 20 SFC per 10^6 ^PBMC is indicated. Box-plots show the median and IQR, and whiskers represent the 10th–90th percentiles. Symbols are specific to each patient according to the key shown in Additional file 1. Randomisation into three groups performed at week 12 is represented by different colours. Group A (red) received placebo, group B (purple) received alternate day dosing of rhGH and group C (blue) received twice weekly dosing of rhGH. Significant p values of <0.05 are shown.

**Figure 5:**
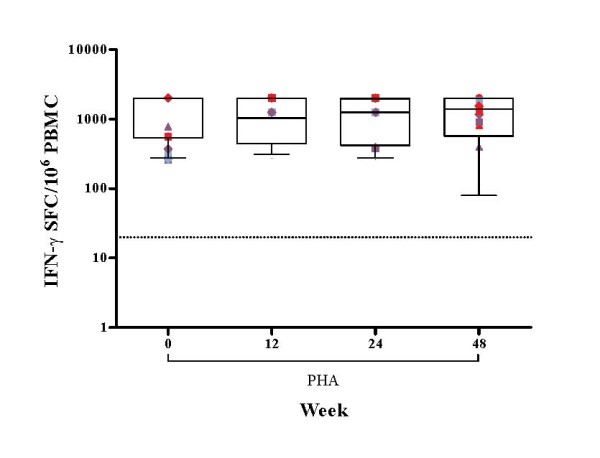
**IFN-γ production by T cells in response to PHA in HAART treated patients at baseline and at weeks 12, 24 and 48. **Results are expressed as the mean number of SFC per 10^6 ^PBMC of duplicate cultures with variation among duplicates <10%. The positive threshold of ≥ 20 SFC per 10^6 ^PBMC is indicated. Box-plots show the median and IQR, and whiskers represent the 10th–90th percentiles. Symbols are specific to each patient according to the key shown in Additional file 1. Randomisation into three groups performed at week 12 is represented by different colours. Group A (red) received placebo, group B (purple) received alternate day dosing of rhGH and group C (blue) received twice weekly dosing of rhGH.

Virus-specific CD8^+ ^T-cell responses were observed at baseline, in the absence of HIV-1-specific CD4^+ ^T-cell responses, in one patient only. Generally, daily administration of rhGH, in the presence of HAART, induced a significant increase in HIV-1-specific CD8^+ ^T-cell responses evaluated with rVV constructs; (Gag rVV p = 0.0059, Pol rVV p = 0.0049) and whole peptide pools of Gag (p = 0.0049) and Pol (p = 0.0025) proteins in IFN-γ ELISpot assays (Figure 4). Such virus-specific CD8^+ ^T-cell responses were maintained at week 24 in all patients including those who received no further rhGH. At week 48, CD8^+ ^virus-specific T-cell responses had significantly declined except those directed at the pool of Pol and Gag peptides (Figure 4). There was no significant change in IFN-γ-secreting responses to mitogenic stimulation throughout the 48-week period (Figure 5).

**Figure 6:**
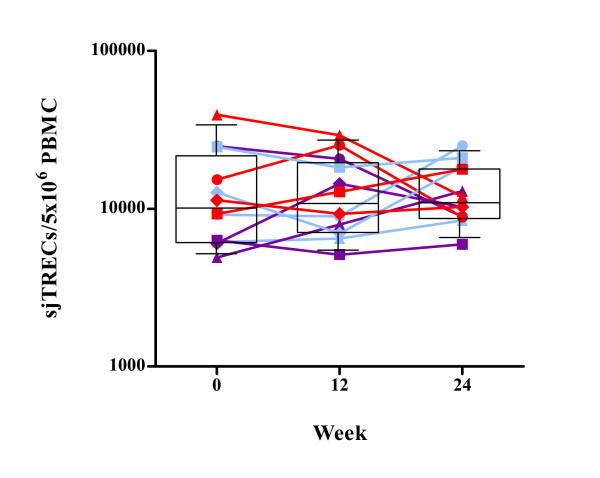
**Signal joint T-cell receptor excision circle (sjTREC) levels over the course of 24 weeks.** The number of sjTRECs/5 × 10^6 ^PBMC was measured, as described in materials and methods, at baseline, weeks 12 and 24 after initiation of rhGH therapy. Box-plots show the median and IQR, and whiskers represent the 10th–90th percentiles. Symbols are specific to each patient according to the key shown in Additional file 1. Randomisation into three groups performed at week 12 is represented by different colours. Group A (red) received placebo, group B (purple) received alternate day dosing of rhGH and group C (blue) received twice weekly dosing of rhGH.

To establish whether rhGH treatment induced changes in thymic T-cell output, TREC levels were analysed in each patient. There was no significant increase in TREC levels between baseline and week 12 or week 24. At baseline, week 12 and week 24 the TREC levels of groups A, B and C were not significantly different from one another. TREC levels, in all three groups, remained constant throughout the course of the 24 week period of growth hormone treatment (Figure 6).

Levels of proviral DNA remained constant throughout the 48-week study with no significant differences observed between the medians at any of the time points, although an increase was seen in three of 12 patients (Table 1). These increases were not observed in patients in whom plasma viral load was detectable (Additional file 1). A negative correlation was observed between the change in anti-Nef CD4^+ ^T-cell proliferative response and change in the level of HIV-1 proviral DNA between baseline and week 12 (r^2 ^= 0.501, p = 0.0148). The change in IFN-γ response mounted against rVV Gag was positively correlated with the change in proviral load between weeks 0 and 12 (r^2 ^= 0.395, p = 0.0383).

**Table 1:** Proviral DNA at baseline (week 0) and weeks 12, 24 and 48 after rhGH immunotherapy.

**Proviral DNA, HIV-1 copy number/μg of total DNA**				
**Patient^a^**	**Baseline**	**Week 12**	**Week 24**	**Week 48**

1 B	ND	8	ND	16
2 C	111	17	ND	46
3 C	476	853	711	625
4 B	536	452	307	220
5 A	55	46	50	52
6 A	223	359	99	93
7 C	315	150	83	238
8 B	42	83	28	57
9 B	91	103	35	56
10 C	15	1212*	750*	ND
11 A	363	3926*	1830*	2277*
12 A	48	27	336*	138

*Mean ± sem*	*206 ± 56.6*	*603 ± 321.3*	*433 ± 178.2*	*347 ± 199.9*
*Median*	*111*	*127*	*203*	*93*
*(Range)*	*(15–536)*	*(8–3926)*	*(28–1830)*	*(16–2277)*

^a ^Randomisation group at week 12 to 24: A – placebo recipients; B – alternate day dosing; and C – twice weekly dosing. ND – Not Done. *Notable increase.

Daily injections of rhGH over a 12-week period induced both CD4^+ ^and CD8^+ ^HIV-1-specific T-cell responses in HAART-treated, chronic infection. However, these benefits are not maintained with less frequent dosing. There was no significant change in thymic output, proviral load, CD4^+ ^T-cell count or plasma viral load as a result of daily rhGH administration in conjunction with HAART up to week 12, or throughout the entire course of the study when dosing was altered according to group. An increased anti-Nef proliferative response was correlated to a decrease in proviral load, and an increased anti-rVV Gag IFN-γ response correlated with an increase in proviral load.

## Discussion

HIV-1-specific CD4^+ ^HTL and CD8^+ ^CTL responses have been shown to inversely correlate with viral replication and disease progression in long-term nonprogressors and protection from productive infection in HIV-1-infected and exposed seronegative individuals [11,12,17,25-27]. Studies in mice have confirmed that dysfunction of CD4^+ ^T cells affects the efficacy of CD8^+ ^T cells in controlling viral replication [28-30]. This forms the basis of understanding HIV-1 immunopathogenesis, as well as providing the rationale for the design of immunotherapeutic strategies aimed at inducing virus-specific CD4^+ ^and CD8^+ ^T-cell responses, in chronic HIV-1 infection [1]. Here we assessed the effects of rhGH therapy on the immune system of chronically HIV-1-infected individuals, in the context of long-term successful HAART. Significant increases in both CD4^+ ^and CD8^+ ^HIV-1-specific T-lymphocyte responses observed after 12 weeks of daily injections of rhGH, concomitantly with HAART, suggest that this combination appears to boost both immune reconstitution and HIV-1-specific T-cell responses in the absence of viral load. This dramatic effect appeared to be particularly focused on HIV-1-specific immunity, as the CD4^+ ^HTL responses to other viral and recall antigens, mitogens and IL-2 were largely unaffected. Statistically significant increases in anti-VZV and -TTox responses were observed, however only 3/12 individuals (anti-VZV) and 5/12 individuals (TTox) exhibited emergence of positive proliferative responses above threshold after receiving rhGH therapy, where the response had been negative at baseline. Significant changes in HSV responses correlate with clinical manifestations, however no significant differences were observed between baseline and week 12. We suggest that, when added to HAART, rhGH immunotherapy may target HIV-1-specific responses because these responses are defective in ways which recall responses are not [11,13,31-36]. Hence rhGH plays a role in resolving the specific defect restricted to HIV-1-specific T cells.

Randomisation of patients into placebo, alternate day dosing or twice weekly dosing at week 12, resulted in a decline in HIV-1-specific CD4^+ ^T-cell responses which occurred in all patients by week 24, which was also apparent at week 48. However, increases in HIV-1-specific CD8^+ ^T-cell responses seen at week 12 were more durable as these were maintained by week 24 regardless of randomisation, and despite the apparent disappearance of HIV-1-specific CD4^+ ^T-cell responses. At week 48, after patients had received HAART alone (no immunotherapy) for the final 24 weeks of the study, HIV-1-specific CD8^+ ^T-cell responses specific to Pol were still above positive threshold for most patients, whereas responses to other HIV-1 antigens had diminished and HIV-1-specific CD4^+ ^T-cell responses remained undetectable.

TREC levels remained unchanged indicating invariable thymic output or alternatively sjTREC levels may reflect increased division rate of naïve T cells as a result of rhGH-induced activation. Previous observations of lower levels of sjTRECs (per 5 × 10^6 ^PBMC) in chronic progressors may not be entirely due to a reduction in thymic function but could be affected by dilution of sjTRECs as a result of immune hyperactivation-induced proliferation in these individuals [22,37]. Assessment of both sjTRECs and βTRECs provides an indication of the extent of intrathymic proliferation [38], which determines the thymic output of naïve T cells [39]. However, the analysis of ratios in these studies has confirmed that sjTREC levels are a good indication of thymic output, and are not hugely influenced by proliferation in the periphery [22,38].

These changes were accompanied by an unexpected notable increase in proviral HIV-1 DNA in 3/12 patients by week 24, which was maintained in only one of the patients tested at week 48. There was a negative correlation observed between the change in anti-Nef proliferative T-cell responses and the change in levels of proviral DNA between weeks 0 and 12. This suggests that targeting HIV-1 Nef with a proliferative T-cell response decreases the amount of proviral DNA/μg total DNA, reducing the number of infected cells/HIV-1 genome copy number per infected cell. A positive correlation was observed between the changes in anti-rVV Gag IFN-γ response and change in proviral DNA levels. Increased breadth and level of anti-HIV-1 Gag IFN-γ responses have previously been shown to be associated with a decrease in plasma viraemia [40], however the effect of anti-Gag IFN-γ responses on the level of proviral DNA has not yet been so comprehensively investigated. A loss of response to Nef has previously been associated with increased opportunistic infection and viral load [41]. We have also observed that a proliferative response to the regulatory HIV-1 protein Nef is preserved in a cohort of HIV controllers, compared to the absence observed in chronic progressors [17]. These findings, in conjunction with the correlation presented here, suggest Nef as a feasible and potentially rewarding target for future research into effective anti-HIV-1 immune responses.

Diminution of CD4^+ ^T-cell responses after daily rhGH was discontinued suggests that these cells may require stronger and/or constant signals from rhGH to recover and provide continuous help. CD8^+ ^T-cell responses may be maintained for a limited period without CD4^+ ^T-cell help [42], but may eventually decline following withdrawal of rhGH therapy. The absence of virus-specific CD4^+ ^T-cell responses, along with a decrease in specific CD8^+ ^T-cell responses by week 48, may reflect exhaustion and/or lack of CD4^+ ^T-cell help to facilitate the complete function of effective HIV-1-specific CD8^+ ^T cells [28-30]. Nevertheless, it is apparent that adequate HIV-1-specific CD4^+ ^T-cell help, provided via immunomodulatory therapy with rhGH has 'kick-started' the appropriate effective anti-HIV-1 CD8^+ ^T cells.

Alternatively, the increase in fully functional HIV-1-specific CD4^+ ^T cells (despite constant CD4^+ ^T-cell counts) may, in some cases, result in *de novo *preferential infection of these cells as previously described [15]. Although this may suggest a risk in the increase of the viral reservoir due to infection of newly formed CD4^+ ^T cells, it might also be indicative that rhGH induces elimination of HIV-1 from its reservoirs. The possible purgative effect of rhGH on HIV-1 from latent reservoirs may result in *de novo *cellular infections possibly accounting for some of the transient pattern of proliferative responses observed in virus-specific CD4^+ ^T-cell responses. This would in turn explain the consistent levels of proviral DNA seen throughout the study. However other explanations cannot be excluded as it is thought that the presence of HAART should prevent or at least minimise *de novo *infection of CD4^+ ^T cells.

We have previously observed an increase in naïve CD4^+ ^and CD8^+ ^T cells, a significant increase in memory/effector CD8^+ ^T cells, as well as recovery of natural killer (NK) cell numbers and function in the same study population of patients shown here [43,44]. Our findings provide novel insights and extend previous observations that daily injections of rhGH for treatment of HIV-1-associated lipodystrophy result in a good outcome [45], as well as having beneficial effects on the immune system such as inducing thymocyte differentiation, improving function of lymphocytes, increasing thymic mass and directly affecting the thymic epithelium [46-49].

It is, however, unlikely that rhGH mediates its effect by increasing antigenic load, since the induction of HIV-1-specific T-cell responses after 12 weeks of rhGH therapy was seen in all patients, despite the unchangeable levels in proviral DNA and undetectable viraemia. When considering the established mechanisms of action of rhGH, it is suggestive that some of the beneficial effects on both the developing thymocytes and thymic stroma may lead to emergence of new CD4^+ ^and CD8^+ ^T cells with anti-HIV-1 potential (i.e. a broader T-cell repertoire). A hypothetical model of the potential effects of rhGH on peripheral lymphocyte dysregulation in HIV-1 infection should, however, also be considered, as it is possible that rhGH therapy reverses some of the negative effects on peripheral responses of PBMC. In this respect, beneficial effects in the periphery may be explained by the ability of rhGH to break the HIV-1-induced T-cell dysfunction/anergy.

Once again, and in a similar fashion to other therapeutic strategies in the HAART setting (i.e. treatment interruption, IL-2 +/- therapeutic immunisation, and IL-2+GM-CSF immunotherapy), removal of immuno- or in this case endocrino-therapy results in loss of induced anti-HIV-1 CD4^+ ^HTL in chronic HIV-1 infection [1,2,13].

Since plasma viral loads remained below the level of detection at all time points, infection of such CD4^+ ^HTL may be the result of very low levels of viral activity undetectable using current viral load assays (<50 copies/ml plasma). Theoretically, effective drug therapy should protect HIV-1-specific CD4^+ ^HTL once they are induced, but this does not seem to be the case since they appear only transiently. Ongoing viral replication in lymph nodes (sanctuary sites, and other reservoirs) may affect the required function of CD4^+ ^HTL needed to subsequently impact on the ability of virus-specific CD8^+ ^CTL to control HIV-1.

Endocrine and cytokine feedback mechanisms may be operational and act beyond the parameters of the immune system. Nevertheless this study underlines the importance of the neuro-endocrine-immunological axis and points out the possibility that hormonal intervention with rhGH might be associated with both *de novo *generation of T lymphocytes (due to increased thymic output) [50,51] as well as increased function of HIV-1-specific T cells (due to restored differentiation/maturation pathways and reversal of anergic dysfunction). In summary, growth hormone immunotherapy and/or antigenic stimulation, concomitant with HAART, may in time induce naïve T cells, IL-2 production and response, CD4^+ ^T-cell proliferation, as well as both induction and maintenance of HIV-1-specific CD8^+ ^T cells. Larger clinical studies/trials are warranted to fully prove clinical value.

## Conclusion

It is known that loss of T-cell function occurs during HIV-1 infection. Here we have provided evidence to demonstrate that rhGH treatment promotes the restoration of T-cell responses against HIV-1, a restoration that declines with cessation of treatment. Since HIV-1^+ ^patients commonly develop growth hormone abnormalities, our data have important implications for the treatment of HIV-1, and raise the possibility that rhGH may form part of an immune-based therapeutic programme tailored to the treatment of HIV-1 disease.

## Consent

Written informed consent was obtained from all subjects.

## Competing interests

The authors declare that they have no competing interests.

## Authors' contributions

All authors have read and approved the final version of this manuscript. NI and GM conceived the study, co-ordinated its design, participated in the application for ethical approval and secured funding for the study. NI was responsible for the overall management of the study and GM undertook patient care and management. NI, AH, SW and JD carried out laboratory work, collected and analysed the data and conducted the transfer and interpretation of the data for final preparation of the manuscript. Statistical analysis was carried out by SW and NI. NI, AH, SW and JD participated in writing the manuscript.

## Supplementary Material

Additional file 1 **Table 1**. Patient characteristics at baseline (week 0), and weeks 12, 24 and 48 of the study. Click here for file
