# Supplementary material for: Effects of recombinant human growth hormone on HIV-1-specific T-cell responses, thymic output and proviral DNA in patients on HAART: 48-week follow-up
Source: J Immune Based Ther Vaccines. 2008 Oct 31;6:7. doi: 10.1186/1476-8518-6-7 (PMC2613878; doi:10.1186/1476-8518-6-7)
Supplement: Additional file 1 — Table 1. Patient characteristics at baseline (week 0), and weeks 12, 24 and 48 of the study. [file 1476-8518-6-7-S1.doc]

**Table 1.** Patient characteristics at baseline (week 0), and weeks 12, 24 and 48 of the study.

|  | | | **Baseline** | | | **Week 12** | | | **Week 24** | | | **Week 48** | | |
| --- | --- | --- | --- | --- | --- | --- | --- | --- | --- | --- | --- | --- | --- | --- |
| Patient^a^ |  | **Age, years** | **CD4 count,**  **cells/μl blood** | **CD8 count,**  **cells/μl blood** | **HIV-1 RNA, copies/ml plasma** | **CD4 count,**  **cells/μl blood** | **CD8 count,**  **cells/μl blood** | **HIV-1 RNA, copies/ml plasma** | **CD4 count,**  **cells/μl blood** | **CD8 count,**  **cells/μl blood** | **HIV-1 RNA, copies/ml plasma** | **CD4 count,**  **cells/μl blood** | **CD8 count,**  **cells/μl blood** | **HIV-1 RNA, copies/ml plasma** |
| 1 B | ● | 40 | 823 | 1239 | <50 | 714 | 1218 | <50 | 688 | 1027 | <50 | 745 | 956 | <50 |
| 2 C | ● | 32 | 436 | 555 | <50 | 422 | 735 | <50 | 456 | 667 | <50 | 418 | 567 | <50 |
| 3 C | ▲ | 38 | 636 | 1235 | 1555 | 616 | 1209 | 5017 | 676 | 1179 | 2027 | 518 | 737 | 2788 |
| 4 B | ▲ | 39 | 604 | 1613 | <50 | 425 | 1137 | <50 | 371 | 899 | <50 | 415 | 980 | <50 |
| 5 A | ● | 45 | 667 | 553 | <50 | 697 | 680 | <50 | 647 | 559 | <50 | 540 | 557 | <50 |
| 6 A | ▲ | 58 | 320 | 421 | <50 | 431 | 837 | <50 | 393 | 917 | <50 | 364 | 750 | <50 |
| 7 C | ⏹ | 41 | 257 | 884 | <50 | 269 | 747 | <50 | 375 | 986 | <50 | 443 | 954 | <50 |
| 8 B | ⏹ | 40 | 622 | 1602 | 17089 | 435 | 776 | <50 | 609 | 1202 | <50 | 577 | 942 | <50 |
| 9 B | **⬥** | 53 | 211 | 1074 | <50 | 217 | 1336 | <50 | 844 | 1369 | <50 | 241 | 884 | <50 |
| 10 C | **⬥** | 40 | 293 | 715 | <50 | 262 | 791 | <50 | 286 | 708 | <50 | ND | ND | ND |
| 11 A | ⏹ | 52 | 374 | 1288 | <50 | 563 | 1624 | <50 | 713 | 2357 | <50 | 599 | 1364 | <50 |
| 12 A | **⬥** | 43 | 498 | 1061 | <50 | 375 | 980 | <50 | 421 | 908 | <50 | 341 | 709 | <50 |
| *Mean±sem* |  | *43.4±2.1* | *478.4±55.6* | *1020±115.6* | *1594±1414* | *452.2±47.7* | *1005.8±85.7* | *465±413* | *550.7±54.5* | *1079.1±147.4* | *229±179.8* | *472.8±42* | *928±108* | *298±249* |
| *Median* |  | *40.5* | *467* | *1067.5* | *9322* | *428* | *908.5* | *5017* | *532.5* | *951.5* | *2027* | *443* | *884* | *2788* |
| *(Range)* |  | *(32-58)* | *(211-823)* | *(421-1613)* | *(<50-17089)* | *(217-714)* | *(680-1624)* | *(<50-5017)* | *(286-844)* | *(559-2357)* | *(<50-2027)* | *(241-745)* | *(557-1364)* | *(<50-2788)* |

^a^ Randomisation group at week 12 to 24: A - placebo recipients; B - alternate day dosing; and C - twice weekly dosing. Changes in CD4 and CD8 T-cell numbers and viral load between different time points are all non-significant. <50 = undetectable. ND – Not Done.
